# An Entropy-Based Automated Cell Nuclei Segmentation and Quantification: Application in Analysis of Wound Healing Process

**DOI:** 10.1155/2013/592790

**Published:** 2013-03-05

**Authors:** Varun Oswal, Ashwin Belle, Robert Diegelmann, Kayvan Najarian

**Affiliations:** ^1^Department of Computer Science, Virginia Commonwealth University, Richmond, VA 23284, USA; ^2^Department of Biochemistry & Molecular Biology, Virginia Commonwealth University, Richmond, VA 23298, USA

## Abstract

The segmentation and quantification of cell nuclei are two very significant tasks in the analysis of histological images. Accurate results of cell nuclei segmentation are often adapted to a variety of applications such as the detection of cancerous cell nuclei and the observation of overlapping cellular events occurring during wound healing process in the human body. In this paper, an automated entropy-based thresholding system for segmentation and quantification of cell nuclei from histologically stained images has been presented. The proposed translational computation system aims to integrate clinical insight and computational analysis by identifying and segmenting objects of interest within histological images. Objects of interest and background regions are automatically distinguished by dynamically determining 3 optimal threshold values for the 3 color components of an input image. The threshold values are determined by means of entropy computations that are based on probability distributions of the color intensities of pixels and the spatial similarity of pixel intensities within neighborhoods. The effectiveness of the proposed system was tested over 21 histologically stained images containing approximately 1800 cell nuclei, and the overall performance of the algorithm was found to be promising, with high accuracy and precision values.

## 1. Introduction

Analysis of microscopy images is one of the most fundamental goals in the realm of immunohistochemistry. The primary tasks involved in the analysis of histologically stained tissue sections are cell nuclei counting, detecting abnormal cell nuclei, and the presence of antigens within the target cells. Results derived from these analyses are most frequently used in the clinical setting to help diagnose a wide spectrum of pathologies. In the past, pathologists accomplished most of these tasks by the means of manual measurements; for example, the quantification of total cells and abnormal cells was performed through manual hand counting. These manual methods are not only time consuming, but the results they yield are often susceptible to inconsistency due to human error. However, as the result of recent advancements in microscopic imaging technology and computational image processing techniques [1], there has been significant growth of research towards translational computational systems that can detect, analyze, classify, and quantify cell nuclei from microscopic images. Adapting to robust automated image processing techniques for primary tasks such as cell nuclei segmentation and quantification will not only prove to be time efficient for pathologists, but these techniques will also be capable of producing consistent results.

In recent years, numerous image processing techniques have been proposed for cell nuclei segmentation [2]. While some techniques only perform the task of cell nuclei segmentation and quantification, techniques that are capable of further detecting and classifying abnormal tumors (cell nuclei) that cause various types of cancer have also been proposed. A cell nuclei segmentation algorithm incorporating unsupervised color clustering, morphological operations, and local thresholding has been proposed to distinguish the cancerous and noncancerous areas in histologically stained images and then segment the clustered cell nuclei [3]. K-means clustering is implemented as unsupervised color clustering technique for cell nuclei segmentation in [4]. Another technique that uses contour detection and contour optimization combined with local gradient information and color deconvolution has been used to detect the optimal threshold values for nuclei segmentation [5]. Entropic-based thresholding methods for cell nuclei segmentation are proposed by Wang and Gudla et al. [6, 7].

A popular technique in the realm of image processing known as region growing is combined with a graph-cuts-based algorithm that incorporates Laplacian of Gaussian (LoG) filtering to detect cell nuclei [8]. Stained endocrine cell nuclei are segmented by a sequential thresholding algorithm that uses a Support Vector Machine (SVM) type of artificial neural network [9]. An adaptive-attention-window-(AAW-) based cell nuclei segmentation technique that exploits quadtree decomposition is proposed by Ko et al. [10]; the size of the AAW dynamically adapts to the region of interest in the input image, and the final cell nuclei segmentation is performed within each AAW. Histogram analysis and optimal local thresholding are followed by morphological procedures to segment a variety of cell nuclei found within the bladder and skin tissue [11]. Watershed segmentation and adaptive thresholding methods are also widely used to achieve automated segmentation of cell nuclei [12–15]. Singh et al. propose the use of a feedforward backpropagation neural network for the classification of the segmented cell nuclei into two categories: benign and malignant breast tumor (nuclei); the proposed neural network is also capable of classifying the detected malignant breast tumor in terms of type 1, type 2, and type 3 [15].

The selection of the above publications exemplifies the wide range of image processing techniques and practical diagnostics applications that encompass the realm of cell nuclei segmentation in immunohistochemistry. The well-established cell nuclei segmentation and differential immunostaining techniques that have proven to be so valuable in the cancer field are now being applied to the field of wound healing research [16]. It is of interest to note that many of the characteristics and cell functions that are manifest in cancer are also found during wound healing [3]. New research strategies to explore human wound healing are now available and allow for the in-depth investigation of the specific cell types that participate in the highly orchestrated events that occur during tissue injury and repair [4, 17]. Development of such translational computation medical systems has been and will be providing invaluable insight into understanding the complex nature of the wound healing process [18, 19].

The vast amount of research in this realm also emphasizes on the need for automated computational systems for cell segmentation techniques that produce accurate and reproducible results. However, the task of cell segmentation is still one of the most challenging tasks in biomedical image processing mainly because the histological specimens that are used for the image acquisition process are 2-dimensional sections of 3-dimensional tissue samples [8]. Images acquired from 2-dimensional histological specimen often contain cells with uneven distribution of color intensities, weak edges, and even incomplete nuclei. These are some key characteristics of microscopic histology images due to which the development of robust automated cell segmentation techniques still remains a challenge. Marker-based watershed segmentation techniques rely on automated detection of marker positions to perform accurate segmentation, however, the task of detecting the number of markers and their positions is not trivial, and over segmentation is often evident in the results. Simple edge detection-based techniques perform well in regions with strong edges but tend to cause over segmentation in regions with weak or poorly defined edges. Active contour or snake methods perform better on cell nuclei with weak edges, but these techniques often require supervision or optimized configuration files with priori information for parameter settings [20]. Hence, there is a need for an automated segmentation system that extracts cell information without requiring any user input.

Thresholding techniques are fairly simple but still effective; they are widely used for the segmentation of histological images because the regions of interest within these images are distinguishable from the other components by visual features such as color and texture [21, 22]. The entropy-based thresholding algorithm presented in this paper uses the color intensity information of pixels and the spatial correlation between pixel intensity values to segment cell nuclei. The proposed technique segments a histological image by classifying it into object (cell nuclei) and background regions; all pixels with intensity values greater (or lesser) than a global threshold value are grouped as the objects, while the remaining pixels are classified as the background. Most of the aforementioned techniques usually perform the cell nuclei segmentation on histological images stained by the Hematoxylin & Eosin (H&E) stain, whereas the proposed entropy-based thresholding algorithm segments cell nuclei from images stained by H&E and three more immunostains for specific cell phenotypes. Endothelial lineage cells were identified by the presence of platelet endothelial cell adhesion molecule cluster of differentiation 31 on the cell surface (CD-31); macrophage functional cells were identified by the presence of a specific cytoplasmic granule found in macrophages called CD-68; and contractile functioning cells were characterized by the presence of intracellular alpha-smooth muscle actin (SMActin).

The following section explains the image acquisition and histological procedures involved in the preparation of the testing dataset.  Section 3 thoroughly explains the methods and mathematical formulae involved in the computation of the proposed technique.  Section 4 presents the results that were obtained by testing the proposed segmentation technique on a dataset of 21 immunohistochemically stained images, and finally conclusions and future work are briefly discussed in Section 5.

## 2. Data Preparation

The dataset used in the testing of the proposed algorithm consists of 21 immunohistochemically stained images. The images in the dataset were acquired from human tissue sections derived from PTFE (expanded polytetrafluoroethylene) tubes that were removed at 5, 7, and 14 days after implantation [5]. The image acquisition process was performed in the described timely manner to characterize the 4 distinct overlapping phases: hemostasis, inflammation, proliferation, and remodeling that occur during the healing process of simple acute wounds. The 4 phases are associated with biological markers and some distinct but overlapping cellular events that can be observed through change in features, such as number of cell nuclei and size of the average nuclei in a tissue section.

The tissue collection and histological staining procedures involved in the preparation of the data set are as follows. Using alcohol and povidone-iodine topical antiseptic, the site of implantation was sterilized and anesthetized using 3 cc lidocaine (1%) without epinephrine. Five, 6.0 cm, of high-porosity PTFE (polytetrafluoroethylene Custom Profile Extrusions, Tempe, AZ) tubes were implanted subcutaneously into the inner aspect of the upper arms of a healthy volunteer subject. Standardized placement was made by a 5.5 cm cannulation of the subcutaneous tissue in a proximal direction. Using a sterile 14-gauge trochar containing PTFE tubing, the skin was punctured, and the trochar was inserted subcutaneously arising through the skin 5.5–6.0 cm away. The trochar was then removed, and the proximal and distal ends of the PTFE tubing were sutured to the skin using a single 5.0 nylon suture. The implantation site was covered with antibiotic ointment and a transparent surgical dressing. On day 14, the PTFE tube was removed and stored in 10% formalin. The wound tissue contained within the fixed PTFE tube was then processed and embedded in paraffin, and 5 micron sections were prepared using standardized histologic techniques. Positive and negative control sections were included to ensure reproducible staining. Hematoxylin & Eosin (H&E) stain was used to highlight the cellular components, and standard immunostaining techniques were used to identify endothelial cells (CD-31), macrophages (CD-68), and contractile cells (*α*-SMActin).

The derived tissue sections were examined using a Zeiss LSM 510 NLO Meta confocal/multiphoton laser scanning microscope. For confocal imaging, the 488, 561, and 633 nm laser lines were used for sample imaging. Images were collected using sequential illumination (i.e., one laser per channel) to avoid signal cross-talk amongst channels. The images were collected using a 63x/1.4 n.a. oil immersion lens (for single photon confocal imaging) or a 63x/1.2 n.a. IR water immersion objective (for multiphoton imaging). The human study was carried out under the approval of the Institutional Review Board of Virginia Commonwealth University, School of Medicine (IRB number 11087).

## 3. Methodology

**Figure 1:**
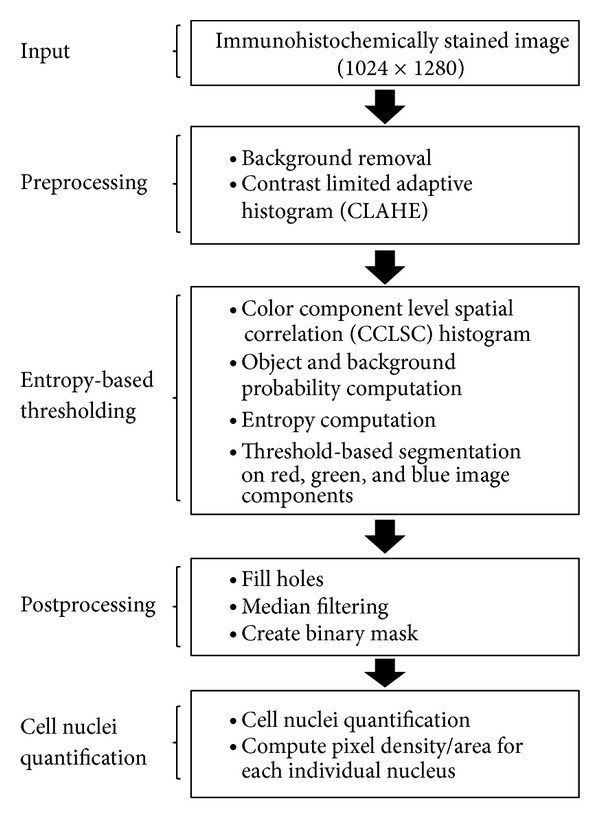
Overview of the proposed algorithm.

This section provides an in-depth explanation of the proposed entropy-based image segmentation technique. The flowchart in Figure 1 illustrates the steps involved in the cell nuclei segmentation process. The proposed algorithm is composed of four steps; image preprocessing, entropy-based thresholding, post-processing, and cell nuclei quantification.

### 3.1. Preprocessing

The preprocessing of an immunohistochemically stained input image *I* begins with a background removal process. The background removal process eliminates all the white space (background) that is captured in image *I* due to the empty spaces present on microscopic slides.

**Figure 2:**
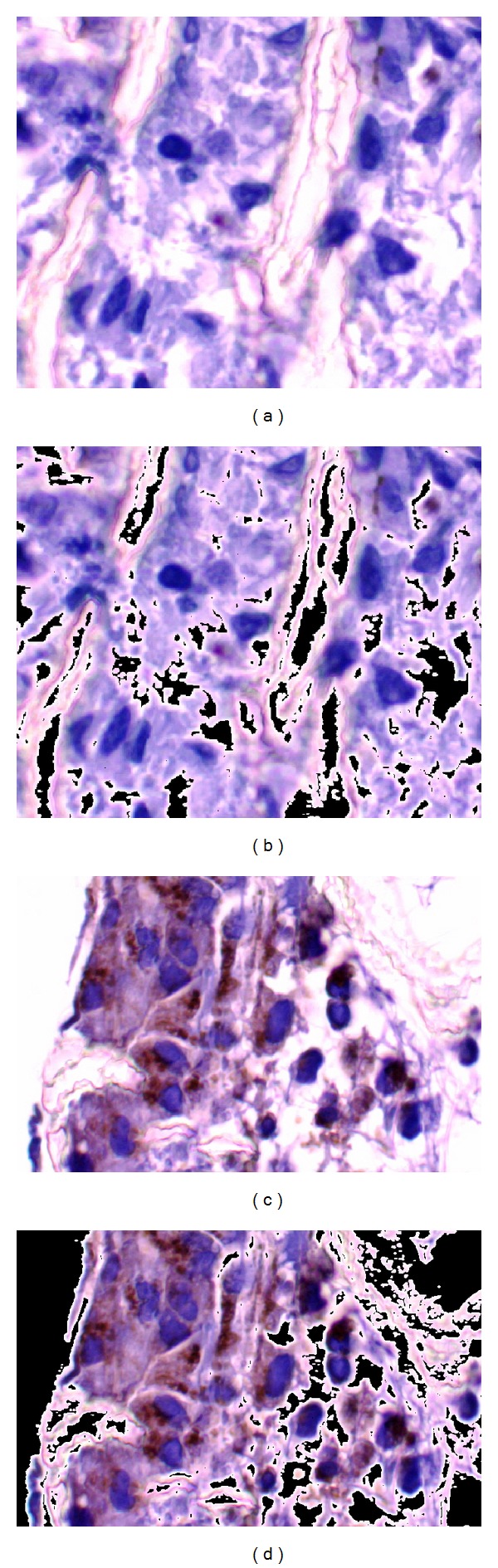
Results from background removal process. (a), (c) Input images. (b), (d) Resulting images composed of *K*_*r*_, *K*_*g*_, and *K*_*b*_ color component images.

Although there are several options with respect to color spaces wherein the processing of the image can be performed, for this project the cell extraction is primarily performed in the RGB color space. Other color spaces such as YCbCr, LAB, and HSV were tested, and in comparison the RGB color space consistently provided the best results. This is because the objective is to extract cell structures based on the color information present within the image, hence distinguishing the different biological objects within the image. The background removal process starts by separating the RGB color image *I* into its red, green, and blue color components to produce 3 color component images *I*_*r*_, *I*_*g*_, and *I*_*b*_, respectively. The local range of the component images *I*_*r*_, *I*_*g*_, and *I*_*b*_ is then computed by finding range values for each individual pixel contained in *I*_*r*_, *I*_*g*_, and *I*_*b*_. The result of this operation yields three output images *J*_*r*_, *J*_*g*_, and *J*_*b*_, in which the value of each output pixel is its local range value, that is, the difference between the maximum and the minimum pixels values within a 3-by-3 neighbourhood surrounding the output pixel. Next, the images *J*_*r*_, *J*_*g*_, and *J*_*b*_ are multiplied together, and the resulting image is then converted to a binary mask *B*. The pixels with value 1 in mask *B* represent the region of interest, and the pixels with value 0 represent the eliminated white space in the input image. Image *B* typically contains noisy components of isolated pixels that are eliminated by median filtering. Finally, the image *B* is individually multiplied to *I*_*r*_, *I*_*g*_, and *I*_*b*_ to produce the three color component images *K*_*r*_, *K*_*g*_, and *K*_*b*_. The RGB image composed of images  *K*_*r*_, *K*_*g*_, and *K*_*b*_ represents only the stained tissue section containing the cell nuclei. The results of the background removal process are illustrated in Figure 2.

A popular histogram equalization technique called Contrast Limited Adaptive Histogram Equalization (CLAHE) is then used in its orignal form to enhance the local contrast in the color component images *K*_*r*_, *K*_*g*_, and *K*_*b*_. Lighting and illumination conditions are very crucial in the acquisition of microscopic images and these conditions are not necessarily the same in every microscopic setup. Different levels of lighting conditions can usually cause differences in the gray-level distribution of pixels in images [23, 24], and therefore, CLAHE is used in the proposed algorithm to uniformly equalize the varying gray-level distributions in any stained immunohistochemical input image.

In CLAHE, contrast enhancement is performed locally in small regions called “tiles”, each tile's histogram is equalized to provide a better overall visual distinction between target objects (cell nuclei) and background (intercellular matter). Additionally, the use of CLAHE ensures that the stained cell nuclei in the tissue section are enhanced uniformly, thereby providing accurate recognition of cell nuclei irrespective of the influences of different staining procedures. The histograms derived from the operation of CLAHE are chosen to maintain a uniform shape. The computation of entropy in the proposed algorithm is based on Shannon's entropy, for which the net information values are calculated within 5-by-5 neighbourhoods throughout the image; the number of tiles for CLAHE's operation is chosen to be close to the total number of 5-by-5 neighbourhoods present in the input image. Once the contrast enhancement is performed on *K*_*r*_, *K*_*g*_, and *K*_*b*_, a series of probability and entropy computations are performed on each component image to determine its optimal threshold value for cell nuclei segmentation.

### 3.2. Entropy-Based Thresholding

After CLAHE is performed on the 3 color component images, the thresholding technique described below is applied to each component image. The proposed entropy-based thresholding method has 3 steps: computation of the color component level spatial correlation (CCLSC) histogram, computation of object and background probabilities, and the computation of object and background entropies. Threshold values for each color component image are obtained once all the calculations are performed. The mathematical computations involved in each step are described in the following sections.

#### 3.2.1. Computation of CCLSC Histogram

The entropy-based thresholding technique relies on the CCLSC histogram which is a modified version of the Grey-level spatial correlation histogram presented in [25]. Two probability distributions are required for the computation of the CCLSC histogram, a histogram distribution of each color component image and a distribution of similarity indices within pixel neighbourhoods that is defined below.

Let *F* denote a color component input image of size *P* × *Q* which has a color intensity value *f*(*x*, *y*) for a pixel located at coordinate (*x*, *y*) in image *F*. The set of all color intensity values is denoted by the set *G* = {0, 1,…, 255}. The similarity index *g*(*x*, *y*) for a pixel located at (*x*, *y*) is computed by determining the number of surrounding pixels that have intensity values within an *ϵ* difference of that pixel's intensity value *f*(*x*, *y*). Similarity indices are computed within *N* × *N* pixel neighbourhoods, where *N* is a positive odd number and *ϵ* is a number between 0 and *N* × *N*. The choice of values for *N* and *ϵ* depends on characteristics of the input images. Smaller values for *N* and *ϵ* work well on images with lower magnifications such as 10x or 20x, and bigger *N* and *ϵ* values work better on images with higher magnification. Values for *N* and *ϵ* are both empirically chosen to be 5 for the implementation of this algorithm. The similarity index uses spatial correlation information of pixel intensity values to preserve important image information such as the edges of cell nuclei. The objects and background regions often tend to have high similarity indices, whereas the features such as edges produce discontinuities in the image's neighbourhoods and are therefore associated with lower similarity indices.

The similarity index is then computed within every pixel neighbourhood, and the degree of similarity is based on the difference of intensity values between the pixel located at the center of the 5 × 5 neighbourhood and all other pixels in the neighbourhood. The lowest similarity count that any neighbourhood can have is 1, that is, a neighbourhood with no other pixel values within an *ε* difference of the center pixel's intensity value will have a similarity index of 1. In any *N* × *N* neighbourhood where *N* is an odd number, the color intensity value of the pixel located at the center of that neighbourhood can be denoted by

(1) $$\begin{array}{c} f\left(\text{center}\right)=f\left(\frac{N+1}{2},\frac{N+1}{2}\right). \end{array}$$

The similarity index *g*(*x*, *y*) for a *N* × *N* neighbourhood is mathematically expressed as

(2) $$\begin{array}{c} g\left(x,y\right)=\sum_{i=1}^{N}\sum_{j=1}^{N}\{\begin{array}{cc} 1, & \text{if}\;\left|f\left(i,j\right)-f\left(\text{center}\right)\right|\leq \epsilon ,\\ 0, & \text{if}\;\left|f\left(i,j\right)-f\left(\text{center}\right)\right|>\epsilon . \end{array} \end{array}$$

The CCLSC histogram is then computed by combining the probability distribution of the histogram of the color component image and the probability distribution of the similarity indices of neighbourhoods. The CCLSC histogram *h*(*k*, *m*) is mathematically defined as

(3) $$\begin{array}{c} h\left(k,m\right)=\text{Prob}\left(f\left(x,y\right)=k,g\left(x,y\right)=m\right), \end{array}$$

where *k* is a value in the set *G* and *m* is a similarity index that can have values in the range {1,…, *N* × *N*}. The normalized CCLSC histogram $\hat{h}\left(k,m\right)$ is then computed by

(4) h^(k,m)=No.  of  pixels  with  f(x,y)=kP×Q∗No.  of  neighbourhoods  with  g(x,y)=mTotal  no.  of  neighbourhoods.

**Figure 3:**
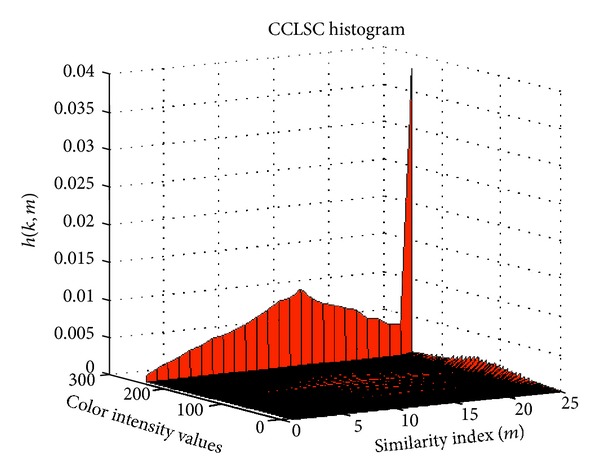
Surface plot of the CCLSC histogram.

Once the CCLSC $\hat{h}\left(k,m\right)$ has been computed, it is used to determine the object and background entropies of the color component image.  Figure 3 shows the surface plot of an instance of the CCLSC histogram.

#### 3.2.2. Object and Background Probability Distributions

The calculation of object and background entropies require probability distributions associated with an image's object and background regions that are derived in the following way. A threshold value *t* is needed to segment an image's object from its background, *t*′s value is chosen such that it partitions the set of color intensities *G* into 2 subsets, *G*_*O*_ and *G*_*B*_. Let *G*_*O*_ = {0, 1, 2,…, *t*} be the set of pixel values that represent the objects, and let *G*_*B*_ = {*t* + 1, *t* + 2, …, 255} be the set of pixel values that represent the background region. The probability distribution for the image's object is expressed as

(5) [h^(0,1)PO(t),…,h^(0,N×N)PO(t),    h^(1,1)PO(t),…,h^(1,N×N)PO(t),…,h^(t,N×N)PO(t)],

and the distribution associated with the background is given by

(6) [h^(t+1,1)PB(t),…,h^(t+1,N×N)PB(t),    h^(t+2,1)PB(t),…,h^(255,N×N)PB(t)],

where

(7) $$\begin{array}{c} P_{O}\left(t\right)=\sum_{k=0}^{t}\sum_{m=1}^{N\times N}\hat{h}\left(k,m\right),\\ P_{B}\left(t\right)=\sum_{k=t+1}^{255}\sum_{m=1}^{N\times N}\hat{h}\left(k,m\right),\\ P_{O}\left(t\right)+P_{B}\left(t\right)=1. \end{array}$$

#### 3.2.3. Computation of Object and Background Entropies

**Figure 4:**
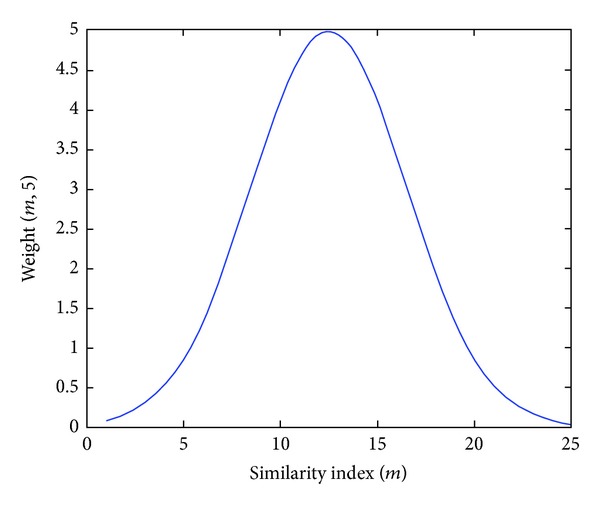
Graph of weight function weight  (*m*, 5).

The probability distributions described in the previous section are used to compute the object and background entropies. According to the principle of Shannon's entropy, the measure of uncertainty from a source equals the net value of information obtained from the source. Features such as noise and edges are associated with higher entropy values because they produce discontinuities between the object and the background which produce more uncertainity in images, that is, net information. As noted in Section 3.2.1, the background and object regions often have higher similarity index values (*m*), whereas edges often have values that lie in the mid range of the set {1,…, *N* × *N*}. A weight function is used in the computation of entropy to assign higher weights to the range of similarity indices that often represent edges of cell nuclei. The weight equation is

(8) $$\begin{array}{c} \text{weight}\left(m,N\right)=5e^{-{\left(m-(N\times N/2)\right)}^{2}/32}, \end{array}$$

where *N* is a positive odd number and *m* is a number in the set {1,…, *N* × *N*}. Figure 4 illustrates the weight function's emphasis in the calculation of the object and background entropy values.

The object entropy *H*_*O*_(*t*, *N*) is computed as

(9) $$\begin{array}{c} H_{O}\left(t,N\right)=-\sum_{k=0}^{t}\sum_{m=1}^{N\times N}\frac{\hat{h}\left(k,m\right)}{P_{O}}\ln\left[\frac{\hat{h}\left(k,m\right)}{P_{O}}\right]\text{weight}\left(m,N\right), \end{array}$$

and the background entropy *H*_*B*_(*t*, *N*) is computed as

(10) HB(t,N)=−∑k=t=1255∑m=1N×Nh^(k,m)PB×ln⁡[h^(k,m)PB]weight(m,N).

After the computation of entropies the function Φ(*t*, *N*) is maximized to yield the optimal threshold value *T* that will be used to segment the input image's target objects (cell nuclei) from the background. The function Φ(*t*, *N*) is expressed as

(11) $$\begin{array}{c} \Phi \left(t,N\right)=H_{O}\left(t,N\right)+H_{B}\left(t,N\right), \end{array}$$

and *T* is given by

(12) $$\begin{array}{c} T=\text{maximum}\left(\Phi \left(t,N\right)\right). \end{array}$$

#### 3.2.4. Thresholding-Based Segmentation

Performing the procedures described in previous section on the preprocessed images *K*_*r*_,  *K*_*g*_, and *K*_*b*_ yields 3 output threshold values *T*_*r*_,  *T*_*g*_, and *T*_*b*_. The 3 threshold values are used to segment the red, green, and blue color pixel components representing cell nuclei in the images *I*_*r*_,  *I*_*g*_, and *I*_*b*_, respectively.

**Figure 5:**
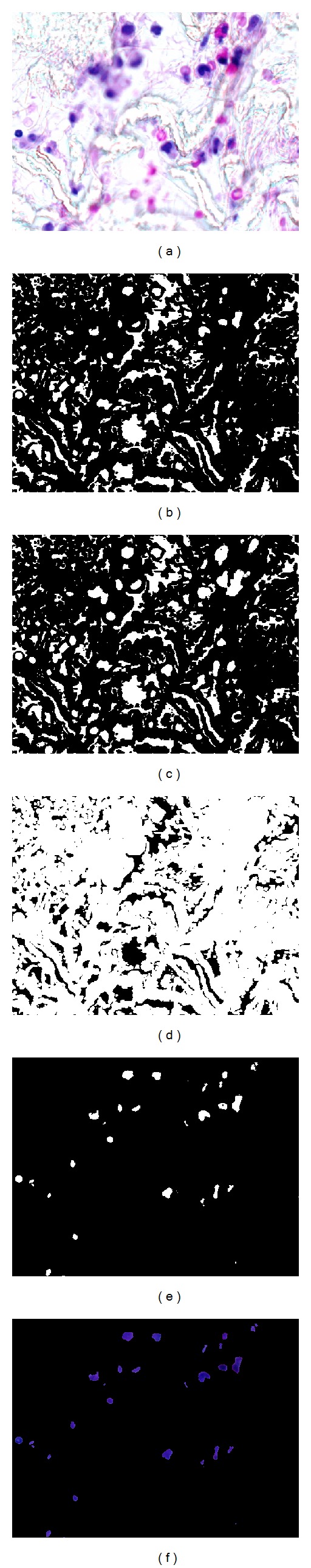
(a) Input image *I*. (b), (c), and (d) Binary images *B*_*r*_, *B*_*g*_, and *B*_*b*_. (e) Binary mask *BW* = (*B*_*r*_∗*B*_*g*_∗*B*_*b*_) of segmented cell nuclei. (f) Segmented cell nuclei in resulting RGB image *R*.

It was experimentally observed that the pixels representing cell nuclei in histologically stained images are composed of lower intensity values in the red and green color component images and higher intensity values in the blue component color image. Therefore, all pixels in *I*_*r*_ and *I*_*g*_ that have intensity values below the output threshold values *T*_*r*_ and *T*_*g*_ are considered to represent cell nuclei, whereas all pixels in *I*_*B*_ that have intensity values greater than *T*_*B*_ are considered to represent the cell nuclei. In the process of segmentation, three binary images *B*_*r*_, *B*_*g*_, and *B*_*b*_ are constructed in which all pixels that are considered as objects in *I*_*r*_, *I*_*g*_, and *I*_*b*_, are valued as 1 at their respective locations within the binary images. The background regions are represented by pixels with value 0. The binary images *B*_*r*_, *B*_*g*_, and *B*_*b*_, are then multiplied together to produce a binary image *BW* that contains only the segmented cell nuclei. In order to obtain the resulting cell nuclei segmentation in its original color, the color component images *I*_*r*_, *I*_*g*_, and *I*_*b*_, are individually multiplied to the binary mask *BW*; the resulting red, green, and blue color component images are then combined to yield an image *R* in RGB color space which contains the results of the segmentation process. The procedures described in this section are illustrated in Figure 5.

### 3.3. Post Processing

The image *R* obtained from the segmentation process contains the extracted cell nuclei, but it also contains unwanted noise that occurs due to similarities in color intensities of cellular and other noncellular regions. The postprocessing procedure attempts to remove most of the unwanted noise so that the results of the cell nuclei quantification contain fewer false positives. The process consists of 2 steps, firstly, a morphological technique called fill-holes is applied on all 3 color component images of *R*. The fill-holes operation is useful in maintaining some structural details of cell nuclei that may have been lost during the process of segmentation.

**Figure 6:**
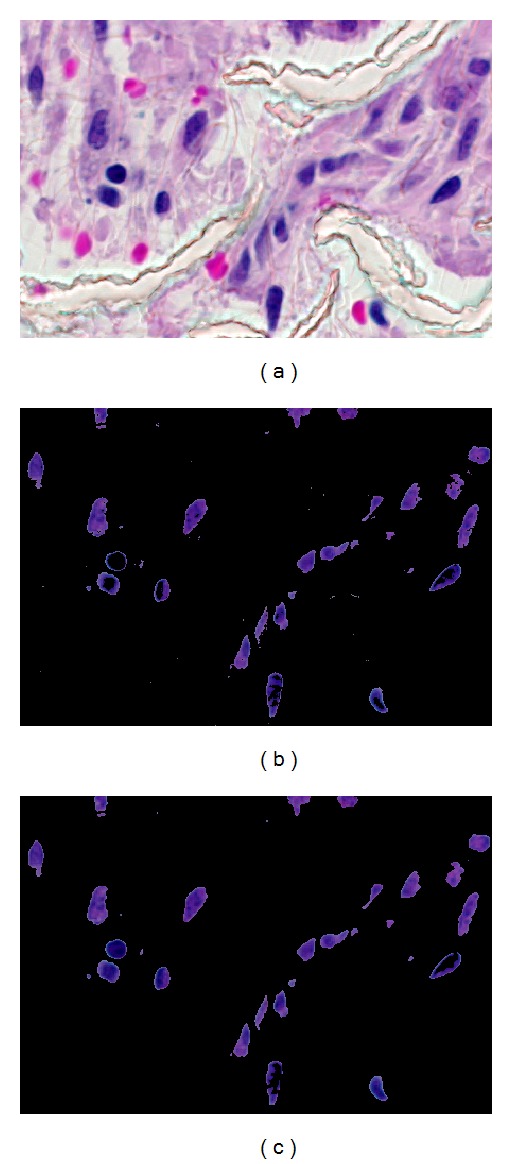
(a) Input image  *I*. (b) Segmented image *R*. (c) Postprocessed image *R*.

The second step in the postprocessing of image *R* is median filtering. Median filtering is a nonlinear operation that is widely used to reduce salt and pepper noise in images. Median filtering is performed on each color component layer of *R*. After the median filtering, the image *R* is denoised and is ready for cell nuclei quantification. Results of the fill-holes and median filtering operations can be observed in Figure 6.

### 3.4. Cell Nuclei Quantification

**Figure 7:**
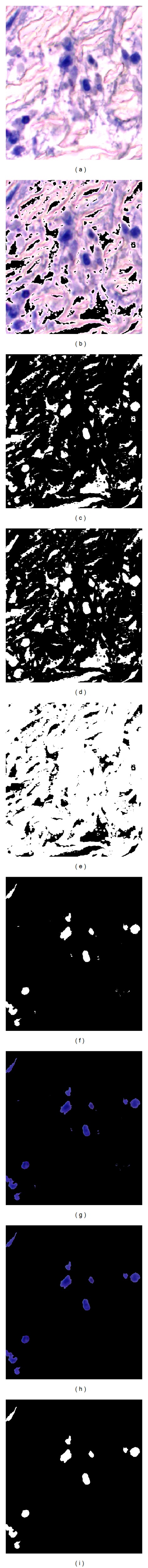
(a) Input image *I*. (b) Background less input image. (c) Red component thresholded at 90 to create image *B*_*r*_. (d) Green component thresholded at 104 to create image *B*_*g*_. (e) Blue component thresholded at 97 to create image *B*_*b*_. (f) Binary image *BW* = (*B*_*r*_∗*B*_*g*_∗*B*_*b*_) of segmented cell nuclei. (g) Image *R* of cell nuclei with minimal noise. (h) Postprocessed image *R* with no noise. (i) Binary mask of output image *R*.

In order to quantify the cell nuclei, the postprocessed image *R* is first converted to a greyscale image and then to a binary image using Otsu's thresholding method [26]. The conversion to grayscale allows the image to be represented in a bimodal fashion so as to obtain Otsu's threshold values. Each connected component of pixels representing a cell's nucleus in the resulting binary image is then counted to yield a total cell nuclei count within an image. The results obtained from the testing of the proposed algorithm are presented in the following section.  Figure 7 illustrates the results of the preprocessing, entropy-based thresholding, and postprocessing steps on an input image.

## 4. Results

The results obtained by testing the proposed automated segmentation technique on a dataset of 21 immunohistochemically stained images are presented in Table 1.

The testing dataset consisted of 21 images belonging to a single patient that were stained using either Hematoxylin & Eosin (H&E) stain, cluster of differentiation 31 (CD-31), cluster of differentiation 68 (CD-68), or alpha-smooth muscle actin (*α*-SMActin). The cell nuclei from 21 test images were manually hand-counted by a pathologist, and the results that were obtained from the manual procedure were compared to the results generated by the automated segmentation technique. The qualitative and quantitative effectiveness of the proposed algorithm's performance is presented by means of its precision, accuracy, sensitivity, and specificity. Images acquired using 40x magnification usually contain more noise components, that is, small groups of connected pixels that do not represent cell nuclei, than the images that are acquired using a 60x magnification. This is due to the fact that the 40x images present a larger area of the tissue section in which the color of the stain is often expressed on small noncellular regions as well. The additional noise affects the precision of the segmentation technique, and this effect can be observed in Table 1 for some 40x images stained by CD-31 and CD-68. Therefore, it is ideal to use images captured at higher magnifications with the proposed algorithm, as they will obtain results with higher accuracy and precision. The true negatives in this study, that is, number of correctly identified noise components, were determined by the difference between the total number of cell nuclei quantified in an image before and after the postprocessing step. The overall accuracy and precision of the proposed segmentation algorithm based on the total number of cell nuclei identified are 95.55% and 91.27%, respectively. The high sensitivity, 96.45%, and the specificity value, 95.07%, achieved by the segmentation technique's performance suggests that the proposed method is effective at segmenting most cell nuclei while accurately identifying, distinguishing, and removing high volumes of noise from the segmented images.

In comparison to a related method for cell segmentation based on shape stability [20], the proposed method outperforms the quality of cell extraction and the precision of count for all cases with the given dataset. To ensure that the proposed system performs well on datasets that have been stained using different procedures, the system was also tested on additional immunohistochemically stained images of cancer cells hosted on the web by groups engaged in biomedical imaging research [27]. The segmentation results closely matched the accuracy and precision that were achieved in the results presented above.

## 5. Conclusion and Future Work

A novel translational computation system for automated cell nuclei segmentation and quantification has been proposed in this paper. Cell nuclei segmentation is a task that has several medical motivations ranging from the detection of malignant cell nuclei (tumor) in cancerous tissue images to the observation of cell nuclei for the characterization of the wound healing process within the human body. The proposed system uses an entropy-based thresholding technique to yield 3 optimal threshold values that are used to segment cell nuclei from images in the RGB color space. The entropy-based computations were based of the concepts introduced in [25]; however, the proposed algorithm introduces new methods such as the background removal preprocessing step, a noise removal postprocessing step, and a modified CCLSC histogram. The proposed technique is consistent in producing highly accurate and precise results of cell nuclei quantification; the technique overcomes the limitations of the existing time-consuming manual cell quantification methods and has great potential for use amongst pathologists. Future work will be directed towards improving the accuracy and precision of the proposed algorithm as well as towards the identification and classification of the various types of cell nuclei such as fibroblasts, and macrophages, which are segmented from the immunohistochemically stained images.

## Figures and Tables

**Table 1:** Results of automated segmentation performance on 21 test images.

Stain	ID/magnification	Manual quantification (cells)	Automated quantification (cells)	Accuracy (%)	Precision (%)	Sensitivity (%)	Specificity (%)
SMActin	Day 5–40x	101	100	92.96	91	90.01	94.67
	Day 5–60x	59	60	91.82	88.33	89.83	93
	Day 7–40x	166	187	93.55	85.56	96.38	92.19
	Day 7–60x	87	94	93.96	89.24	95.40	93.10
	Day 14–40x	85	88	92.75	89.77	92.94	92.62

H&E	Day 5–40x	86	86	100	100	100	100
	Day 7–40x	141	138	99.05	100	97.87	100
	Day 7–60x	66	71	95.10	90.14	96.96	94.06
	Day 14–40x	105	108	98.69	97.22	100	97.6
	Day 14–60x	42	38	95.28	100	88.095	100

CD-31	Day 5–40x	76	77	99.38	98.70	100	98.85
	Day 5–60x	52	49	97.43	100	94.23	100
	Day 7–40x	140	188	94.32	77.77	97.22	93.66
	Day 7–60x	61	59	97.61	100	95.08	100
	Day 14–40x	98	104	97.97	94.23	100	97.97
	Day 14–60x	67	67	100	100	100	100

CD-68	Day 5–40x	99	96	91.36	76.19	96.96	89.39
	Day 5–60x	68	66	95.13	84.61	97.05	94.54
	Day 14–40x	143	138	98.55	100	96.50	100
	Day 14–60x	69	70	99.49	98.57	100	99.23

Total		1811	1884
Average				95.55	91.27	96.45	95.07
